# A cross-sectional study investigating the relationships between self-management abilities, productive patient-professional interactions, and well-being of community-dwelling frail older people

**DOI:** 10.1007/s10433-020-00586-3

**Published:** 2020-10-23

**Authors:** Lotte Vestjens, Jane Murray Cramm, Anna Petra Nieboer

**Affiliations:** Erasmus School of Health Policy & Management, Department of Socio-Medical Sciences, Erasmus University Rotterdam, P.O. Box 1738, 3000 DR Rotterdam, The Netherlands

**Keywords:** Well-being, Self-management, Productive patient-professional interactions, Primary care, Frail older people, Cross-sectional design

## Abstract

Worldwide, the maintenance of well-being in ageing populations with associated frailty has become increasingly important. To maintain well-being during ageing, investment in frail older people’s self-management abilities and the fostering of productive interactions with healthcare professionals may lead to higher levels of well-being. The aim of this study was to investigate the relationships between community-dwelling frail older people’s self-management abilities, productive patient-professional interactions and well-being, while controlling for socio-demographic characteristics. This cross-sectional study included 588 community-dwelling frail older people (aged ≥ 75 years) from 15 general practitioner (GP) practices in the Netherlands. Well-being (Social Production Function Instrument for the Level of well-being short), productivity of interactions with GPs (relational coproduction instrument), and self-management abilities (Self-Management Ability Scale short) were measured during in-home face-to-face interviews by trained interviewers. Data were analysed using descriptive statistics, correlation analyses, and linear mixed-effects models. Significant relationships were detected between self-management abilities and the overall, social, and physical well-being of older people, and between productive interactions with GPs and overall and social well-being, but not physical well-being. In a time of ageing populations with associated frailty, investment in frail older people’s self-management abilities and the productivity of patient-professional interactions may be beneficial for this population’s well-being.

## Introduction

Worldwide, the maintenance of ageing populations’ well-being has become increasingly important (Steptoe et al. 2015). Frailty, defined as the presence of problems or losses in multiple domains (physical, psychological, and social) of human functioning (Gobbens et al. 2010b), is associated with lower levels of well-being among community-dwelling older people (Andrew et al. 2012). Compared with the general population, frail older people have a compromised ability to realise and maintain well-being (Nieboer and Cramm 2018). This is due to changes and declines in available physical and social resources, and in opportunities to realise well-being (Steverink 2014). Consequently, maintaining the well-being of a frail population is a key challenge (Steptoe et al. 2015). To maintain well-being levels during ageing, investment in frail older people’s self-management abilities and the fostering of productive interactions with healthcare professionals may lead to higher levels of well-being.

Individuals are motivated to improve their living situations to optimise their levels of well-being, although this endeavour is not always successful (Steverink 2014). The balance between resource gains and losses changes over the life span, with losses gradually dominating (Steverink et al. 1998). Consequently, as people grow older, the maintenance of need fulfilment and management of losses therein become increasingly important (Steverink et al. 2005; Steverink 2014). The realisation and maintenance of well-being depend on the possession of adequate resources that aid the fulfilment of needs contributing to well-being, and, more importantly, the ability to manage these resources (Steverink 2014). Self-management abilities consist of a diverse repertoire of cognitive and behavioural abilities to manage resources for fulfilling well-being needs and managing losses (Steverink et al. 2005; Steverink 2014). Older people with better overall self-management abilities are expected to be more effective in creating, maintaining, and restoring their well-being (Steverink et al. 2005; Steverink 2014).

In addition, healthcare professionals can support a person’s development and maintenance of abilities that enable well-being in older age. Researchers and practitioners increasingly recognise the need for person-centred approaches that are responsive to frail older people’s preferences and needs (beyond physical health and clinical outcomes) and are successively aimed at protecting their well-being (WHO 2015). Productive interactions between frail older people and their healthcare professionals (Gittell and Douglass 2012; Gittell 2002, 2006; Wagner et al. 2001) are assumed to be essential in enhancing care processes and optimising (abilities to maintain) well-being (Barr et al. 2003; Nolte and McKee 2008; Wagner et al. 2001, 2005; WHO 2015). The quality of interactions is assumed to affect a person’s well-being. The recognition of a person’s needs may improve patient-professional interactions by encouraging trust and affection (Kuipers et al. 2019), and may provide insight into unfulfilled needs and the associated changes required to protect a frail older person’s well-being (Steverink and Lindenberg 2006).

Previous research has shown that greater self-management abilities are associated with greater well-being among older people (Cramm et al. 2012a, b; 2013a, b; Goedendorp and Steverink 2017; Steverink and Lindenberg 2008). Likewise, research has shown that the productivity of interactions is associated with the improved well-being of chronically ill patients (Cramm and Nieboer 2015b; Kuipers et al. 2019). To our best knowledge, the relationship between productive patient-professional interactions and well-being has not been investigated in a population of independently living frail older people (75 years and older) in a primary care setting in the Netherlands. The primary care setting is considered to be among the most important settings for the delivery of care and support to community-dwelling frail older people (Cesari et al. 2016), with gatekeeping general practitioners (GPs) as central actors in Dutch primary care (van Campen et al. 2013; Kroneman et al. 2016). The aim of this study was to investigate the relationships between community-dwelling frail older people’s self-management abilities, productive patient-professional interactions and well-being, while controlling for socio-demographic characteristics.

### Theories of well-being, self-management, and productive interactions

#### Well-being of frail older people

Social production function (SPF) theory holds that individuals are active producers of their own subjective or psychological well-being via attempts to obtain universal needs of physical and social well-being (Lindenberg and Frey 1993; Lindenberg 1996; Nieboer and Cramm 2018; Ormel et al. 1999, 1997). Overall well-being is considered to be the joint production of physical and social well-being (Ormel et al. 1999, 1997). The SPF theory asserts that the production of physical well-being requires the fulfilment of 2 instrumental needs: comfort (the satisfaction of physical needs and absence of stimuli that create discomfort, e.g. pain and hunger) and stimulation (an adequate level of physical and mental activation, e.g. pleasant levels of physical effort, excitement, and arousal). Social well-being is achieved by obtaining status (a person’s relative ranking, e.g. the sense of being respected and having valued resources), affection (being loved for who one is, irrespective of one’s actions or status, e.g. the feeling of being liked, loved, and accepted, provided mainly in caring relationships), and behavioural confirmation (the sense of doing the ‘right’ thing according to oneself or relevant others e.g. the sense of being useful and doing good things) (Lindenberg 1996; Ormel et al. 1999; Steverink 2014). Each of these instrumental needs can be realised by (multifunctional) means, that is, by activities and endowments. For example, intimate ties contribute significantly to a person’s affection level (Ormel et al. 1999).

### Self-management of well-being

Self-management abilities aid the effective achievement, maintenance, and restoration of physical and social well-being, ultimately leading to the realisation of overall subjective well-being (Steverink et al. 2005; Steverink 2014). Overall self-management ability is defined as ‘a generative capacity (consisting of several sub-abilities) to take care of one’s own important resources, that is, resources that contribute to well-being’ (Steverink and Lindenberg 2008:182). The premise of this conceptualisation is that behavioural and cognitive abilities are connected to the dimensions of well-being (i.e. comfort, stimulation, status, affection, and behavioural confirmation) (Steverink et al. 2005; Steverink and Lindenberg 2008). According to the self-management of well-being (SMW) theory (Steverink et al. 2005), the core interrelated and mutually reinforcing self-management abilities are cognitive abilities (self-efficacy beliefs and having a positive frame of mind), active motivational abilities (taking initiative and investment behaviour), and resource combining abilities (multifunctionality of resources and variety in resources). (1) Self-efficacy beliefs refer to a person’s belief in his or her competence to effectively achieve goals and realise aspects of well-being; and (2) having a positive frame of mind entails the ability to have a positive perspective on the future instead of focusing on losses. In addition, (3) taking initiative reflects a person’s self-motivation to realise aspects of well-being in contrast to being passive or dependent, and (4) investment behaviour refers to the ability to invest in resources for the long-term. Finally, (5) multifunctionality of resources refers to the simultaneous contribution of resources and activities to multiple aspects of well-being in a mutually reinforcing way, and (6) variety in resources refers to the contribution of multiple resources and activities to single aspects of well-being. Although each ability is important on its own, the strengthening of all interacting abilities results in improved self-management for the realisation or maintenance of resources to satisfy well-being needs later in life (Steverink and Lindenberg 2008).

### Productivity of interactions

People try to achieve universal well-being needs by actively producing essential means (realising instrumental needs, e.g. sufficient comfort and affection) in the light of available resources and constraints (Lindenberg 2013; Ormel et al. 1999; Steverink and Lindenberg 2008). Especially for frail older people with disabilities, illnesses, and functional limitations, goal attainment and continued participation in important activities are facilitated by individual relationships and other resources, and can reduce or avoid the deterioration of well-being (Cramm and Nieboer 2016b; Nieboer 2013). To realise needs that promote well-being, care should centre on a person’s preferences, needs, values, and goals (Greene et al. 2012; Rathert et al. 2013; Wagner et al. 2005). Persons partnering with (teams of) healthcare professionals who promote participation in managing life situations, focus on goals relevant to the maintenance of well-being, and provide effective (self-management) support and follow-up are more likely to achieve better outcomes (Bergeson and Dean 2006; Wagner et al. 2005). Consequently, productive patient-professional interactions are assumed to be essential in co-producing the best possible patient outcomes, including well-being (Barr et al. 2003; Wagner et al. 2001, 2005; WHO 2015). Productive interactions between professionals and patients are characterised by accurate, frequent, timely, and problem-solving communication. Effective communication is supported by relationships based on mutual respect, and high levels of shared goals and knowledge, and vice versa (Batalden et al. 2016; Gittell 2012; Gittell and Douglass 2012). The maintenance or improvement of frail older people’s well-being is more likely to be realised when patient-professional interactions are characterised by effective communication and high-quality relationships (Batalden et al. 2016; Gittell 2012; Gittell and Douglass 2012).

## Data and methods

### Study design and setting

This cross-sectional study included GP practices in western North Brabant Province, the Netherlands, and was conducted from mid-2014 to mid-2015. Fifteen of 17 GP practices approached agreed to participate. This study is part of a large-scale evaluation of proactive, integrated primary care for community-dwelling frail older people, which has been described in detail elsewhere (Vestjens et al. 2018).

### Participants and inclusion

The study sample consisted of community-dwelling frail older people (aged ≥ 75 years). Recruitment of this sample was conducted in 2 steps. First, the frailty of all 3545 older people (aged ≥ 75 years) registered at the 15 GP practices was assessed using a postal questionnaire which included the 15-item Tilburg Frailty Indicator (TFI) (Gobbens et al. 2010a). The TFI is a self-report user-friendly questionnaire used to assess frailty in the physical, psychological, and social domains; persons with scores ≥ 5 (range, 0–15) are considered to be frail (Gobbens et al. 2010a). Reminders were sent by mail and telephone to non-responders. A response rate of 83.4% (*n* = 2956) was achieved. As the TFI may not fully encompass all essential aspects of frailty, its use in isolation is not recommended (van Dijk 2015). Therefore, persons whose TFI scores did not indicate frailty (TFI score < 5), could also be identified as frail based on additional examinations or interviews by healthcare professionals. Second, the sample of frail older people derived from the screening (TFI and/or additional frailty examination by healthcare professionals) was assessed by GPs and researchers on eligibility criteria for study participation. We excluded (**1**) frail older people living in nursing homes or homes for older people, (**2**) people with estimated life expectancies of < 3 months, and (**3**) people who were not able to communicate in Dutch. Furthermore, GPs assessed whether reasonable grounds to suspect incapacity to participate and/or to give consent existed (e.g. due to cognitive problems), and people were excluded in such cases. Of 834 potential participants, 588 persons were willing to participate in this study (70.5% response rate).

### Data collection

To collect data, interviewers administered the questionnaires during in-home face-to-face interviews. The interviewers lived in western North Brabant Province and had backgrounds in healthcare; they were trained to conduct the interviews. On average, interviews lasted 60–75 min.

### Measures

#### Well-being

Well-being was measured using the short version of the validated social production function instrument for the level of well-being (SPF-ILs) (Nieboer et al. 2005). This 15-item instrument measures overall well-being, as well as levels of social (behavioural confirmation, status, and affection) and physical (comfort, and stimulation) well-being (Nieboer and Cramm 2018; Nieboer et al. 2005; Ormel et al. 1999, 1997). Answers to the questions are given on a 4-point scale ranging from 1 (never) to 4 (always), and mean scores are calculated. Higher scores indicate greater well-being (Nieboer et al. 2005). The instrument has been shown to provide a reliable and valid assessment of social and physical well-being among older people (Nieboer and Cramm 2018; Nieboer et al. 2005). The Cronbach’s alpha value for overall well-being measured with the SPF-ILs in this study was 0.84, indicating a high degree of reliability. Cronbach’s alpha values for social and physical well-being were 0.80 and 0.77, respectively.

### Productive patient-professional interactions

Frail older people’s perceptions of the productivity of interactions were measured using the validated relational coproduction instrument (Gittell 2000, 2012; Gittell et al. 2000, 2013). In this study, productivity of interactions with GPs was assessed. The relational coproduction instrument consists of 7 survey questions assessing dimensions of communication (frequency, timeliness, accuracy, and problem-solving nature) and relationships (mutual respect, shared goals, and shared knowledge). Together, these dimensions form the productive interaction construct (Gittell et al. 2000, 2012, 2013). The 7 items are rated on a 5-point scale ranging from 1 (never) to 5 (always), and mean scores are calculated. Higher scores represent higher-quality interactions with the GP, as perceived by frail older people. The Cronbach’s alpha value for the relational coproduction instrument in this study was 0.86, indicating a high degree of reliability.

### Self-management abilities

The self-management abilities of frail older people were measured using the short version of the self-management ability scale (SMAS-S) (Cramm et al. 2012a, b; Schuurmans et al. 2005). This 18-item questionnaire assesses a diverse repertoire of self-management abilities for the maintenance of physical and social well-being. The SMAS-S assesses cognitive abilities (self-efficacy beliefs and a positive frame of mind), active-motivational abilities (taking initiative and investment behaviour), and resource-combining abilities (multifunctionality of resources and variety in resources) (Cramm et al. 2012a, b; Schuurmans et al. 2005). Mean SMAS-S scores range from 1 to 6, with higher scores indicating better self-management abilities. The Cronbach’s alpha value for the SMAS-S in this study was 0.91, indicating a high degree of reliability.

### Socio-demographic variables

The questionnaire contained items regarding the persons’ age, sex, educational level, marital status, and (multi)morbidity. Morbidities were indicated on a list of 17 conditions, including diabetes, Chronic obstructive pulmonary disease, heart failure, and hearing disorders. Educational level (elementary school or less and more than elementary school), marital status (married/living together and single/widowed/divorced), and (multi)morbidity (0 or 1 condition and ≥ 2 conditions) were dichotomised.

### Ethical considerations

The medical research ethics committee of the Erasmus Medical Centre in Rotterdam, the Netherlands, reviewed the research proposal (study protocol number MEC-2014–444) and determined that the rules laid out in the Medical Research Involving Human Subjects Act did not apply. Frail older people were informed by telephone and during in-home visits about the study (e.g. purposes, procedures, confidentiality, and contact information for the researchers and interviewers). In addition, participants received a leaflet containing relevant research information. Written informed consent to participate in the study was obtained from all participants.

### Statistical analyses

The socio-demographic characteristics of study participants were analysed using descriptive statistics. Bivariate associations between the study variables (self-management abilities, productive interactions, and well-being) were analysed using pearson correlation coefficients. Linear mixed-effects models (588 frail older people nested in 15 GP practices) were employed to investigate relationships of self-management abilities and productive interactions with GPs to well-being (social, physical and overall). A random intercept was used on the GP practice level. The outcome estimates were adjusted for socio-demographic characteristics (age, sex, educational level, marital status, and multimorbidity). Social, physical and overall well-being served as the dependent variables, and the productivity of interactions and self-management abilities served as independent variables. Assumptions of linear models (including linearity, normality, multicollinearity, homoscedasticity, and significant outliers) were tested and no large violations were found. In addition, we found no indication of a mediating effect between the variables (Hayes 2018). Results were interpreted as significant when two-sided *p*-values were < 0.05. The software package IBM SPSS (version 24 for Windows; IBM Corporation, Armonk, NY, USA) was used for all statistical analyses.

## Results

Table 1 shows descriptive statistics for the socio-demographic characteristics of the study sample, well-being, self-management abilities, and the productivity of interactions with GPs. Of the 588 participants, 68.5% were women, 61.7% were single, and 38.4% had low educational levels. Their mean age was 82.32 (standard deviation (SD), 5.19; range, 75–98) years. Almost 90% of the frail older people reported multimorbidity ( ≥ 2 conditions). The mean SPF-ILs score for overall well-being was 2.640 (SD, 0.492; range, 1–4). Mean scores for physical and social well-being were 2.578 (SD, 0.615; range, 1–4) and 2.678 (SD, 0.553; range, 1–4), respectively. The mean SMAS-S score for self-management abilities was 3.670 (SD, 0.879; range, 1–6) and the mean score for the productivity of interactions with GPs was 3.78 (SD, 1.144; range, 1–5).

**Table 1:** Descriptive statistics for socio-demographic characteristics, well-being, self-management abilities, and productive interactions among frail older people, *N* = 588

	Mean ± SD (range) or *n* (%)	*n*
Age (years)	82.32 ± 5.19 (75–98)	588
Sex (women)	403 (68.5%)	588
Marital status (single)	363 (61.7%)	588
Educational level (low)	226 (38.4%)	588
Multimorbidity ( ≥ 2 diseases)	523 (89.6%)	588
Overall well-being	2.640 ± 0.492	578
Physical well-being	2.578 ± 0.615	581
Social well-being	2.678 ± 0.553	570
Self-management abilities	3.670 ± 0.879	583
Productive interactions with GPs	3.783 ± 1.144	576

Table 2 shows the correlations among self-management abilities, the productivity of interactions with GPs, and well-being. Significant correlations were found between self-management abilities and overall (*r* = 0.701), physical (*r* = 0.589), and social (*r* = 0.603) well-being (all *p* < 0.001). Significant weak correlations were found between the productivity of interactions with GPs and overall (*r* = 0.162) and social (*r* = 0.225) well-being (both *p* < 0.001), but not with physical well-being (*p* = 0.603). Self-management abilities were correlated weakly with the productivity of interactions with GPs (*r* = 0.126, *p* < 0.01).

**Table 2:** Pearson correlations among self-management abilities, productive interactions, and well-being among frail older people, *N* = 588

	Physical well-being	Social well-being	Overall well-being
	*r*	*r*	*r*
Self-management abilities	0.589***	0.603***	0.701***
Productive interactions with GPs	0.022	0.225***	0.162***

^***^p < 0.001 (two-tailed)

Table 3 displays the results of the linear mixed-effects models. Analyses controlled for socio-demographic characteristics revealed significant relationships between self-management abilities and overall, physical, and social well-being (all *p* < 0.001). They also revealed significant relationships between the productivity of interactions with GPs and overall (*p* < 0.05) and social (*p* < 0.001) well-being, but not physical well-being (*p* = 0.212).

**Table 3:** Relationships between self-management abilities, productive interactions, and well-being while controlling for socio-demographic characteristics, as revealed in linear mixed-effects models, among frail older people, *N* = 588

	Physical well-being		Social well-being		Overall well-being	
	*n* = 571		*n* = 560		*n* = 568	
	B	SE	B	SE	B	SE
Constant	0.720	0.367	0.954**	0.337	0.701**	0.264
Age (years)	0.012**	0.004	0.003	0.004	0.006*	0.003
Sex (women)	0.081	0.047	0.047	0.044	0.084*	0.034
Marital status (single)	0.051	0.042	−0.033	0.038	−0.003	0.034
Educational level (low)	0.019	0.042	−0.022	0.038	−0.014	0.030
Multimorbidity ( ≥ 2 diseases)	−0.164*	0.067	−0.066	0.061	−0.125**	0.048
Self-management abilities	0.376***	0.025	0.360***	0.023	0.391***	0.017
Productive interactions with GPs	−0.022	0.018	0.073***	0.016	0.032*	0.013

^*^p < 0.05, **p < 0.01, ***p < 0.001 (two-tailed)

## Discussion

This study aimed to investigate the relationships between community-dwelling frail older people’s self-management abilities, productive patient-professional interactions and well-being, while controlling for socio-demographic characteristics. The study shows that self-management abilities were related significantly to physical, social, and overall well-being in this study sample. The productivity of interactions with GPs was related significantly to social and overall well-being, although the effect sizes were small.

### Well-being and self-management

The finding indicating relationships between self-management abilities and well-being in a sample of community-dwelling frail older people underlines the importance of strengthening these abilities. This is done to manage resources to maintain well-being, and to effectively avoid or cope with losses, later in life (Steverink et al. 2005). The relative difficulty of fulfilling well-being needs increases with age as the availability of resources and opportunities to satisfy needs alters and declines (Steverink et al. 1998; Steverink 2014). In the process of ageing, reserves and resources in several life domains decline, and losses in one domain can reinforce resource-loss in other domains, necessitating the possession of adequate and diverse self-management abilities (Steverink et al. 2005). According to Frieswijk and colleagues (2006), frail older people with deficits in multiple domains may benefit from interventions to improve general self-management abilities aimed at maintaining all aspects of well-being, instead of single target (health) problems (Frieswijk et al. 2006). Dutch governmental policies aim to enhance self-sufficiency and independent living in the community for as long as possible (de Klerk et al. 2019; van Campen et al. 2017), which makes the effective self-management of well-being even more important.

### Well-being and productive patient-professional interactions

The present study showed that productive interactions with GPs in the primary care setting were related significantly to the social well-being and overall well-being (the joint production of physical and social well-being) of community-dwelling frail older people, even after controlling for self-management abilities (although the effect sizes were small); no significant relationship with physical well-being was found. This finding is in line with those from a recent cross-sectional study among patients with multimorbidity in the Netherlands. This study showed that productive interactions with healthcare professionals (GPs, nurse practitioners, and specialists) were related significantly to social well-being, but not physical well-being (Kuipers et al. 2019). The productivity of patient-professional interactions as measured with the relational coproduction instrument (Gittell et al. 2000), consists largely of social aspects (e.g. quality of the patient-professional relationship based on mutual respect, and high levels of shared goals and knowledge) and may thus relate mainly to social well-being goals (Kuipers et al. 2019). In addition, a study by Nieboer and Cramm (2018) has shown that frail older people report lower physical well-being levels compared with a general sample of community-dwelling older people. Frail older people reported lower comfort and stimulation levels, which serve as resources for physical well-being (Nieboer and Cramm 2018). As frailty is related to developing adverse health outcomes (e.g. disability, falls, and hospitalisation) (Vermeiren et al. 2016), frail older people may experience more difficulties with physical well-being. It may be more difficult to affect physical well-being through the quality of the patient-professional relationship and communication. The productivity of interactions with GPs explains only a small part of well-being; other factors contributing to older people’s well-being include personal resources (Pinquart and Sörensen 2000) and neighbourhood characteristics (e.g. social cohesion) (Cramm et al. 2013a, b; Cramm and Nieboer 2015c; Oswald et al. 2011). Although the effect sizes in our study were small, our findings suggest that productive patient-professional interaction may be a resource for the maintenance of well-being and prevention of a decline in needs contributing thereto when facing age-related changes in physical, psychological, and social domains (Williams et al. 2007).

GPs may contribute significantly to their frail older patients’ social and overall well-being outcomes by investing in productive interactions with them. Effective communication between healthcare professionals and frail older people should therefore not focus solely on biomedical and psychosocial domains but should include emotional and affective care (Williams et al. 2007). A trusting patient-professional relationship can be considered to be central in the care process and may be therapeutic for patients, especially frail older people with multimorbidity (Williams et al. 2007). However, widespread problems with communication and collaboration between patients and healthcare professionals have been reported (Øvretveit 2012). Suboptimal patient-professional communication involves healthcare professionals’ failure to create environments and relationships that enable effective communication, suboptimal communication skills, patients’ withholding of information, and healthcare professionals’ failure to provide (understandable) information during consultations or about medications (Øvretveit 2012). Problems with patient-professional collaboration include non-attendance of scheduled appointments, time constraints with respect to consultations, a lack of continuity with healthcare professionals, and the under-involvement of patients in decision-making processes (Øvretveit 2011, 2012). The findings of our study imply the need for healthcare professionals to invest in the quality of communication and relationships with frail older people. To enhance the productivity of interactions, frail older people need to be informed and activated; to whatever degree possible, they need to have goals and plans to protect or improve their health and well-being. To become active partners and wise decision-makers in their care processes, frail older people need high-quality information, and adequate skills, motivation, and confidence to manage their conditions and well-being effectively. They need to understand the importance of information sharing and their own roles in managing their health and satisfying well-being needs. For interactions to be productive, healthcare professionals should be organised, equipped, and trained to conduct productive interactions with frail older people. They need relevant expertise, time, resources and patient information (Bodenheimer et al. 2002a, b; Wagner et al. 1996, 2001, 2005). The support of frail older people in protecting (the potential loss of) well-being requires relational competence to consider their preferences, needs, values and goals, empathise with their situations, and respect their needs and choices (Cramm and Nieboer 2012, 2015b).

### Study limitations

Several limitations of this study should be considered. First, the cross-sectional design limited the investigation of causal relationships. The relationships of self-management abilities and productive interactions to well-being may be dynamic, and longitudinal research of these relationships among community-dwelling frail older people is recommended. Second, the study population was derived from a single province in the Netherlands, which may hamper the generalisability of our findings to other areas and populations of older people. Third, no information was available from non-responders in the study. Non-response to (postal) questionnaires may introduce bias (Edwards et al. 2002); for example, frailty may have been higher among non-responders. Fourth, an integral perspective on frailty as defined by Gobbens and colleagues (2010c) was employed in which physical, psychological, and social domains of human functioning are incorporated and operationalised in the multidimensional TFI (Gobbens et al. 2010c). There is, however, still considerable uncertainty about an internationally recognised and comprehensive definition of frailty (Bergman et al. 2007; Brown and Covinsky 2018; Dent et al. 2016). Disagreements continue about what conceptual frailty approaches should be adopted (Hoogendijk et al. 2019), and instruments used to assess frailty are based on different conceptualisations of the phenomenon. Dominating perspectives in the field include a frailty phenotype in which frailty is defined as a biological syndrome (Fried et al. 2001) or a multifactorial perspective on frailty by the accumulation of health deficits (Mitnitski et al. 2001; Rockwood and Mitnitski 2007). Increasingly, research on frailty stresses the need for a multidimensional perspective (Dury et al. 2018) in which not only physical aspects dominate but the contribution of multiple domains is taken into account (e.g. psychological, social, cognitive, and environmental) (De Witte et al. 2013; Gobbens et al. 2010c; Gustafsson et al. 2012; Markle-Reid and Browne 2003). Based on the continuous debate on defining frailty and its measurement, the TFI may not fully encompass all relevant aspects. However, the TFI is frequently used in the Netherlands and other countries in Europe (Op het Veld et al. 2019). The psychometric properties of the TFI have shown to be good (i.e. good internal consistency and construct validity) (Gobbens et al. 2010a, 2020; Metzelthin et al. 2010). A systematic review of Sutton and colleagues (2016) comparing multicomponent frailty assessment tools has shown that the TFI has the most robust evidence supporting its reliability and validity.

Fifth, other potentially important determinants of (relationships among) self-management abilities, productive interactions, and well-being were not investigated. For example, the quality of care delivery has been shown to be a significant determinant of self-management abilities (Cramm and Nieboer 2015a) and productive patient-professional interactions among chronically ill patients (Cramm and Nieboer 2014) and community-dwelling frail older people (Vestjens et al. 2019). In addition, research of Dury and colleagues (2018) has shown that frail older people possess balancing factors for frailty (i.e. resources to fulfil psychological, social, physical, environmental, and/or cognitive challenges). Balancing factors were present at the individual (e.g. resilience), environmental (e.g. neighbourhood characteristics), and macro level (e.g. financial income), and might contribute to dealing effectively with frailty and increase positive outcomes, such as maintaining well-being. Also, negative and positive turning points and life events such as death of the partner or birth of a grandchild might affect their frailty and outcomes (Dury et al. 2018). In the current study, balancing factors were not explicitly considered, although multiple balancing factors may (partly) overlap or interact with, for example, self-management abilities (e.g. abilities and resources to stay positive or invest in social contacts). Sixth, moderate associations found between frail older people’s self-management abilities and their well-being may be explained (partly) by the use of the SMW theory (Steverink et al. 2005), which is based on the SPF theory (Lindenberg 1996). The core abilities specified in the SMW theory form the construct of self-management ability and are linked explicitly to the dimensions of well-being proposed in the SPF theory (Steverink et al. 2005). Finally, only the productivity of interactions with the GPs was examined, not those with other healthcare professionals in the primary care setting such as elderly care physicians and home care nurses. GPs serve a gatekeeping function and are central actors in primary care (van Campen et al. 2013; Kroneman et al. 2016). Other studies have shown that interactions with GPs tend to be more productive than those with other healthcare professionals (Cramm and Nieboer 2015b, 2016a). This may be explained by the central role of GPs in the Dutch primary care system and the nature of their relationships with older people. GPs are among the most frequently contacted healthcare professionals in primary care, and they often have long histories with their patients (Jansen et al. 2012; Kroneman et al. 2016). These factors may provide more opportunities for the strengthening of relationships and communication between GPs and frail older people. Further investigation of the productivity of interactions with other healthcare professionals is recommended.

### Conclusions

It can be concluded that self-management abilities and productive patient-professional interactions are related to the well-being of community-dwelling frail older people in the Netherlands. In a time of ageing populations with associated frailty, investment in self-management abilities and productive patient-professional interactions in GP practices is expected to be beneficial for the well-being of frail older people.

## Data Availability

The datasets generated and analysed during the current study are not publicly available, but are available from the corresponding author on reasonable request.

## References

[CR1] Andrew MK, Fisk JD, Rockwood K (2012). Psychological well-being in relation to frailty: a frailty identity crisis?. Int Psychogeriatr.

[CR2] Barr VJ, Robinson S, Marin-Link B, Underhill L, Dotts A, Ravensdale D, Salivaras S (2003). The expanded chronic care model: an integration of concepts and strategies from population health promotion and the chronic care model. Hosp Q.

[CR3] Batalden M, Batalden P, Margolis P, Seid M, Armstrong G, Opipari-Arrigan L, Hartung H (2016). Coproduction of healthcare service. BMJ Qual Saf.

[CR4] Bergeson SC, Dean JD (2006). A systems approach to patient-centered care. JAMA.

[CR5] Bergman H, Ferrucci L, Guralnik J, Hogan DB, Hummel S, Karunananthan S, Wolfson C (2007). Frailty: an emerging research and clinical paradigm-issues and controversies. J Gerontol A Biol Sci Med.

[CR6] Bodenheimer T, Wagner EH, Grumbach K (2002). Improving primary care for patients with chronic illness. JAMA.

[CR7] Bodenheimer T, Wagner EH, Grumbach K (2002). Improving primary care for patients with chronic illness: The chronic care model, part 2. JAMA.

[CR8] Brown RT, Covinsky KE (2018). Frailty as an outcome in geriatrics research: not ready for prime time?. Ann Intern Med.

[CR11] Cesari M, Prince M, Thiyagarajan JA, De Carvalho IA, Bernabei R, Chan P, Gutierrez-Robledo LM, Michel J, Morley JE, Ong P, Rodriguez Manas L, Sinclair A, Won CW, Beard J, Vellas B (2016). Frailty: an emerging public health priority. J Am Med Dir Assoc.

[CR14] Cramm JM, Nieboer AP (2012). Relational coordination promotes quality of chronic care delivery in Dutch disease-management programs. Health Care Manage Rev.

[CR15] Cramm JM, Nieboer AP (2014). A longitudinal study to identify the influence of quality of chronic care delivery on productive interactions between patients and (teams of) healthcare professionals within disease management programmes. BMJ Open.

[CR16] Cramm JM, Nieboer AP (2015). Chronically ill patients' self-management abilities to maintain overall well-being: what is needed to take the next step in the primary care setting?. BMC Fam Pract.

[CR17] Cramm JM, Nieboer AP (2015). The importance of productive patient-professional interaction for the well-being of chronically ill patients. Qual Life Res.

[CR18] Cramm JM, Nieboer AP (2015). Social cohesion and belonging predict the well-being of community-dwelling older people. BMC Geriatr.

[CR19] Cramm JM, Nieboer AP (2016). The changing nature of chronic care and coproduction of care between primary care professionals and patients with COPD and their informal caregivers. Int J Chronic Obstr Pulm Dis.

[CR20] Cramm JM, Nieboer AP (2016). Is "disease management" the answer to our problems? No! Population health management and (disease) prevention require "management of overall well-being". BMC Health Serv Res.

[CR12] Cramm JM, Hartgerink JM, de Vreede PL, Bakker TJ, Steyerberg EW, Mackenbach JP, Nieboer AP (2012). The relationship between older adults' self-management abilities, well-being and depression. Eur J Ageing.

[CR21] Cramm JM, Strating MMH, de Vreede PL, Steverink N, Nieboer AP (2012). Validation of the self-management ability scale (SMAS) and development and validation of a shorter scale (SMAS-S) among older patients shortly after hospitalisation. Health Qual Life Outcomes.

[CR13] Cramm JM, Hartgerink JM, Steyerberg EW, Bakker TJ, Mackenbach JP, Nieboer AP (2013). Understanding older patients' self-management abilities: functional loss, self-management, and well-Being. Qual Life Res.

[CR22] Cramm JM, van Dijk HM, Nieboer AP (2013). The importance of neighborhood social cohesion and social capital for the well-being of older adults in the community. Gerontol.

[CR47] de Klerk M, Verbeek oudijk D, Plaisier I, den Draak M,  (2019). Zorgen voor thuiswonende ouderen. Kennissynthese over de zorg voor zelfstandig wonende 75-plussers, knelpunten en toekomstige ontwikkelingen.

[CR23] De Witte N, Gobbens R, De Donder L, Dury S, Buffel T, Schols J, Verté D (2013). The comprehensive frailty assessment instrument: development, validity and reliability. Geriatr nurs.

[CR24] Dent E, Kowal P, Hoogendijk EO (2016). Frailty measurement in research and clinical practice: a review. Eur J Intern Med.

[CR26] Dury S, Dierckx E, van der Vorst A, Van der Elst M, Fret B, Duppen D, Hoeyberghs L, De Roeck E, Lambotte D, Smetcoren AS, Schols J, Kempen G, Zijlstra GAR, De Lepeleire J, Schoenmakers B, Verté D, De Witte N, Kardol T, De Deyn PP, Engelborghs S, De Donder L (2018). Detecting frail, older adults and identifying their strengths: results of a mixed-methods study. BMC Public Health.

[CR27] Edwards P, Roberts I, Clarke M, DiGuiseppi C, Pratap S, Wentz R, Kwan I (2002). Increasing response rates to postal questionnaires: systematic review. BMJ.

[CR28] Fried LP, Tangen CM, Walston J, Newman AB, Hirsch C, Gottdiener J, Seeman T, Tracy R, Kop WJ, Burke G, McBurnie MA (2001). Frailty in older adults: evidence for a phenotype. J Gerontol A Biol Sci Med Sci.

[CR29] Frieswijk N, Steverink N, Buunk BP, Slaets JP (2006). The effectiveness of a bibliotherapy in increasing the self-management ability of slightly to moderately frail older people. Patient Educ Couns.

[CR30] Gittell JH (2000). Organizing work to support relational co-ordination. Int J Human Res Manag.

[CR36] Gittell JH (2002). Relationships between service providers and their impact on customers. J Serv Res.

[CR33] Gittell JH, Kyriakidou O, Özbilgin MF (2006). Relational coordination: coordinating work through relationships of shared goals, shared knowledge and mutual respect. Relational perspectives in organizational studies: A research companion.

[CR31] Gittell JH, Cameron KS, Spreitzer GM (2012). New directions for relational coordination theory. The Oxford handbook of positive organizational scholarship.

[CR32] Gittell JH, Douglass A (2012). Relational bureaucracy: structuring reciprocal relationships into roles. Acad Manag Rev.

[CR34] Gittell JH, Fairfield KM, Bierbaum B, Head W, Jackson R, Kelly M, Laskin R, Lipson S, Siliski J, Thornhill T, Zuckerman J (2000). Impact of relational coordination on quality of care, postoperative pain and functioning, and length of stay. A nine-hospital study of surgical patients. Med Care.

[CR35] Gittell JH, Godfrey M, Thistlethwaite J (2013). Interprofessional collaborative practice and relational coordination: improving healthcare through relationships. J Interprof Care.

[CR38] Gobbens RJJ, van Assen MALM, Luijkx KG, Wijnen-Sponselee MT, Schols JMGA (2010). The Tilburg Frailty Indicator: psychometric properties. J Am Med Dir Assoc.

[CR39] Gobbens RJJ, Luijkx KG, Wijnen-Sponselee MT, Schols JMGA (2010). In search of an integral conceptual definition of frailty: opinions of experts. J Am Med Dir Assoc.

[CR40] Gobbens RJ, Luijkx KG, Wijnen-Sponselee MT, Schols JM (2010). Towards an integral conceptual model of frailty. J Nutr Health Aging.

[CR37] Gobbens RJ, Boersma P, Uchmanowicz I, Santiago LM (2020). The tilburg frailty indicator (TFI): new evidence for its validity. Clin Interv Aging.

[CR41] Goedendorp MM, Steverink N (2017). Interventions based on self-management of well-being theory: pooling data to demonstrate mediation and ceiling effects, and to compare formats. Aging Ment Health.

[CR42] Greene SM, Tuzzio L, Cherkin D (2012). A framework for making patient-centered care front and center. Perm J.

[CR43] Gustafsson S, Edberg AK, Dahlin-Ivanoff S (2012). Swedish health care professionals’ view of frailty in older persons. J Appl Gerontol.

[CR44] Hayes AF (2018). Introduction to mediation, moderation, and conditional process analysis.

[CR45] Hoogendijk EO, Afilalo J, Ensrud KE, Kowal P, Onder G, Fried LP (2019). Frailty: implications for clinical practice and public health. Lancet.

[CR46] Jansen D, Spreeuwenberg P, Heijmans M (2012). Ontwikkelingen in de zorg voor chronisch zieken.

[CR48] Kroneman M, Boerma W, van den Berg M, Groenewegen P, de Jong J, van Ginneken E (2016). The Netherlands: health system review. Health Syst Transit.

[CR49] Kuipers SJ, Cramm JM, Nieboer AP (2019). The Importance of patient-centered care and co-creation of care for satisfaction with care and physical and social well-being of patients with multi-morbidity in the primary care setting. BMC Health Serv Res.

[CR50] Lindenberg S, Lindenberg S, Ganzeboom HBG (1996). Continuities in the theory of social production functions. Verklarende sociologie: opstellen voor Reinhard Wippler.

[CR51] Lindenberg S, Wittek R, Snijders TAB (2013). Social rationality, self-regulation and well-being: the regulatory significance of needs, goals, and the self. Handbook of rational choice social research.

[CR52] Lindenberg S, Frey BS (1993). Alternatives, frames, and relative prices: a broader view of rational choice theory. Acta Sociol.

[CR53] Markle-Reid M, Browne G (2003). Conceptualizations of frailty in relation to older adults. J Adv Nurs.

[CR54] Metzelthin SF, Daniëls R, van Rossum E, de Witte L, van den Heuvel WJ, Kempen GI (2010). The psychometric properties of three self-report screening instruments for identifying frail older people in the community. BMC Public Health.

[CR55] Mitnitski AB, Mogilner AJ, Rockwood K (2001). Accumulation of deficits as a proxy measure of aging. Sci World J.

[CR56] Nieboer AP (2013). Houdbare zorg en ondersteuning in tijden van crisis [Sustainable care in a time of crisis].

[CR57] Nieboer AP, Cramm JM (2018). How do older people achieve well-being? Validation of the social production function instrument for the level of well-being-short (SPF-ILs). Soc Sci Med.

[CR58] Nieboer AP, Lindenberg S, Boomsma A, van Bruggen AC (2005). Dimensions of well-being and their measurement: The SPF-IL Scale. Soc Indic Res.

[CR59] Nolte E, McKee M (2008). Caring for people with chronic conditions: a health system perspective.

[CR60] Op het Veld LPM, Beurskens AJHM, de Vet HCW, van Kuijk SMJ, Hajema KJ, Kempen GIJM, van Rossum E (2019) The ability of four frailty screening instruments to predict mortality, hospitalization and dependency in (instrumental) activities of daily living. Eur J Ageing 16(3):387–394 10.1007/s10433-019-00502-4 PMC6728401 31543731

[CR62] Ormel J, Lindenberg S, Steverink N, Von Korff M (1997). Quality of life and social production functions: a framework for understanding health effects. Soc Sci Med.

[CR61] Ormel J, Lindenberg S, Steverink N, Verbrugge LM (1999). Subjective well-being and social production functions. Soc Indic Res.

[CR63] Oswald F, Jopp D, Rott C, Wahl H (2011). Is aging in place a resource for or risk to life satisfaction?. Gerontol.

[CR64] Øvretveit J (2011). Does clinical coordination improve quality and save money?.

[CR65] Øvretveit J (2012). Do changes to patient-provider relationships improve quality and save money? A review of the evidence about value improvements made by changing communication, collaboration and support for self-care.

[CR66] Pinquart M, Sörensen S (2000). Influences of socioeconomic status, social network and competence on subjective well-being in later life a meta-analysis. Psychol Aging.

[CR67] Rathert C, Wyrwich MD, Boren SA (2013). Patient-centered care and outcomes: a systematic review of the literature. Med Care Res Rev.

[CR68] Rockwood K, Mitnitski AB (2007). Frailty in relation to the accumulation of deficits. J Gerontol A Biol Sci Med Sci.

[CR69] Schuurmans H, Steverink N, Frieswijk N, Buunk BP, Slaets JP, Lindenberg S (2005). How to measure self-management abilities in older people by self-report, the development of the SMAS-30. Qual Life Res.

[CR70] Steptoe A, Deaton A, Stone AA (2015). Subjective wellbeing, health, and ageing. Lancet.

[CR71] Steverink N, Pachana NA, Laidlaw K (2014). Successful development and ageing: theory and intervention. The Oxford Handbook of Clinical Geropsychology.

[CR75] Steverink N, Lindenberg S (2006). Which social needs are important for subjective well-being? What happens to them with aging?. Psychol Aging.

[CR72] Steverink N, Lindenberg S (2008). Do good self-managers have less physical and social resource deficits and more well-being in later life?. Eur J Ageing.

[CR73] Steverink N, Lindenberg S, Ormel J (1998). Towards understanding successful ageing: patterned change in resources and goals. Ageing Soc.

[CR74] Steverink N, Lindenberg S, Slaets JP (2005). How to understand and improve older people’s self-management of wellbeing. Eur J Ageing.

[CR76] Sutton JL, Gould RL, Daley S, Coulson MC, Ward EV, Butler AM, Nunn SP, Howard RJ (2016). Psychometric properties of multicomponent tools designed to assess frailty in older adults: a systematic review. BMC Geriatr.

[CR9] van Campen C, Broese van Groenou M, Deeg D, Iedema J (2013). Met zorg ouder worden. Zorgtrajecten van ouderen in tien jaar.

[CR10] van Campen C, Iedema J, Broese van Groenou M, Deeg D (2017). Langer zelfstandig. Ouder worden met hulpbronnen, ondersteuning en zorg.

[CR25] van Dijk HM (2015). Neighbourhoods for ageing in place.

[CR77] Vermeiren S, Vella-Azzopardi R, Beckwée D, Habbig AK, Scafoglieri A, Jansen B, Bautmans I (2016). Frailty and the prediction of negative health outcomes: a meta-analysis. J Am Dir Assoc.

[CR78] Vestjens L, Cramm JM, Birnie E, Nieboer AP (2018). Evaluating an integrated primary care approach to improve well-being among frail community-living older people: a theory-guided study protocol. BMC Geriatr.

[CR79] Vestjens L, Cramm JM, Nieboer AP (2019). Quality of primary care delivery and productive interactions among community-living frail older persons and their general practitioners and practice nurses. BMC Health Serv Res.

[CR81] Wagner EH, Austin BT, Von Korff M (1996). Organizing care for patients with chronic illness. Milbank Quat.

[CR80] Wagner EH, Austin BT, Davis C, Hindmarsh M, Schaefer J, Bonomi A (2001). Improving chronic illness care: translating evidence into action. Health Aff.

[CR82] Wagner EH, Bennett SM, Austin BT, Greene SM, Schaefer JK, Von Korff M (2005). Finding common ground: patient-centeredness and evidence-based chronic illness care. J Altern Complement Med.

[CR83] WHO (2015). WHO global strategy on people-centred and integrated health services.

[CR84] Williams SL, Haskard KB, DiMatteo MR (2007). The therapeutic effects of the physician-older patient relationship: effective communication with vulnerable older patients. Clin Interv Aging.

